# The significance of task shifting for surgeons in high-income countries: an examination of the Japanese case

**DOI:** 10.3389/fpubh.2024.1435001

**Published:** 2025-01-08

**Authors:** Michioki Endo, Yasuhiro Mizuno, Tsuyoshi Nemoto, Ayu Yasui, Kenji Gonda, Akihiko Ozaki

**Affiliations:** ^1^Department of Surgery, Hyogo Prefectural Awaji Medical Center, Sumoto, Japan; ^2^Marru-Clinic Yokosuka, Yokosuka, Japan; ^3^Department of Home Medical Care, Kashima Kose Hospital, Minamisoma, Fukushima, Japan; ^4^Department of Breast and Thyroid Surgery, Tokiwakai Jyoban Hospital, Iwaki, Japan

**Keywords:** aging, surgeon age, career, primary care, high-income countries

## 1 Introduction

The surgical workforce in high-income countries is experiencing a demographic shift toward an aging population. As of 2019, 16% of surgeons in Canada were over 65 years of age, while this proportion was 9% in the United Kingdom (2018) and 25.7% in Japan (2020) (1). Moreover, in the United States, 51% of surgeons were over the age of 55 as of 2021 (2).

While age-related physical and cognitive decline occurs in surgeons (3), the relationship between surgeon age and surgical outcomes shows mixed results. Although pancreatic resections by surgeons over 60 show higher mortality rates (OR 1.67, 95% CI: 1.12–2.49) compared to those aged 40–50 (4), a large Ontario study (*n* = 1.16 million) found improved outcomes with increasing surgeon age (5). These contrasting findings suggest that decisions about continuing surgical practice should be individualized, considering each surgeon's specific capabilities and circumstances rather than age alone.

The management of aging surgeons' careers varies globally, from mandatory retirement ages (65 years in India, 60/55 years in China/Russia for male/female doctors, 70 years in Pakistan, 67 years in Italy) to no age restrictions (United States) (6). The trend is shifting away from universal retirement ages toward individualized competency assessments, though a standardized, evidence-based approach combining technical and non-technical skills evaluation remains needed to balance patient safety with surgical expertise (6–8). Current assessment programs (*n* = 21), predominantly in the USA (90%), require re-evaluations every 1–5 years, with most programs conducting biennial reviews (7). While most programs include physical (86%) and cognitive (81%) assessments, clinical performance (29%) and record-keeping (14%) evaluations are less common, and none incorporate simulation testing (7).

An underexplored aspect of this discourse is the development of programs supporting surgeons' transition to alternative career paths. The Japanese healthcare system offers valuable insights, where surgeons routinely perform multiple clinical roles including medication management, palliative care, and primary care services (9). This integrated practice model naturally facilitates the transition from surgical to primary care roles, potentially providing a framework for career transition programs globally.

## 2 Case studies from the Japanese healthcare system

To illustrate transition pathways in Japan, we present three case studies. The first features a 76-year-old surgeon who practiced general surgery until age 72. Notably, this surgeon's decision to discontinue surgical practice was not driven by physical limitations, but rather by concerns about long-term follow-up care for cancer patients—a distinctive aspect of Japanese healthcare where surgeons traditionally provide ongoing post-operative care. He currently manages his former surgical patients in a primary care capacity, demonstrating a successful transition model.

The second case highlights Dr. Mizuno, who leveraged his gastroenterological surgery expertise to establish a clinic at age 40, combining endoscopic procedures with comprehensive primary care services. He became particularly renowned for his work in *Helicobacter pylori* eradication in children (10), and now serves on the *H. pylori* treatment guideline committee of the Japanese Society of Gastroenterology. His clinic specializes in *H. pylori* screening for pediatric and adolescent populations, demonstrating how surgical expertise can be effectively translated into specialized primary care practice.

The third case describes Dr. Nemoto, who transitioned from general surgery to primary care at age 47, responding to acute healthcare needs in Fukushima's coastal regions following the 2011 Great East Japan Earthquake (11, 12). Now at 59, he provides palliative and home-based medical care—services that are increasingly vital not only in Fukushima's coastal areas, where aging has accelerated post-disaster (13), but throughout Japan's rapidly aging society (14).

## 3 Discussion

In this case series, we have presented three examples of successful transitions from surgery to primary care. While each surgeon had different motivations and circumstances for transitioning from surgery to primary care, a key commonality was that they all adapted their practice to meet local community needs. Crucially, the surgeon's positive mindset toward such career transitions is fundamental for successful adaptation.

However, as Mizuno notes, the transition from surgical practice to primary care presents several challenges, particularly in reacquiring competencies in pharmacotherapy and patient management. While informal mentorship has proven effective, more structured support systems are essential. While the Japan Surgical Society is working to address the critical nationwide shortage of surgeons—with over 50% of teaching hospitals reporting deficits even in major cities—there is insufficient support for surgeons seeking to transition to other specialties (9). The Ministry of Health, Labor and Welfare in Japan plans to promote generalist physicians by encouraging experienced doctors to retrain as general practitioners, offering supportive programs to help them acquire comprehensive medical skills and incentivizing their relocation to underserved areas, in response to Japan's uneven distribution of healthcare providers (15).

Internationally, although some support programs exist for such career transitions, they remain limited. The Royal Australasian College of Surgeons (RACS) has established a Senior Surgeons Section that serves as a support network and advocacy group for senior surgeons. This section focuses on addressing the professional, financial, and psychological implications of surgical practice transition (16). In Canada, while formal surgical transition programs are still in development, there have been calls for structured programs to support surgeons transitioning their practice (17). A survey of Canadian surgeons highlighted the need for better retirement planning resources and career transition support (17).

Demographic trends in high-income countries are driving increased demand for primary care services. United States projections indicate that by 2034, healthcare demands for the population aged 65 and older will necessitate 407,300 surgeons, concurrent with an anticipated deficit of up to 48,000 primary care physicians (18). Japan's experience as one of the world's most aged societies offers valuable insights for other nations confronting similar demographic transitions. The strategic redeployment of aging surgical expertise through role transition may provide a model for addressing these challenges.

Alternative career pathways for aging surgeons extend beyond primary care practice. Significant contributions can be made through:

1. Medical education: Integration into surgical training programs, simulation centers, and medical schools.
2. Research and quality improvement: Application of clinical expertise to surgical outcomes research and protocol development.
3. Administrative leadership: Transition to healthcare policy and hospital administration roles.
4. Surgical coaching: Mentorship and technical guidance for early-career surgeons.

Thus, their extensive experience can be leveraged to make diverse contributions beyond clinical practice, encompassing various domains of healthcare advancement.

Future research priorities should encompass the development and evaluation of structured retraining programs, establishment of competency assessment protocols, and outcome measurement across various career transition pathways. These efforts should be complemented by the establishment of comprehensive support systems for aging surgeons, including structured career counseling, formal mentorship programs, and flexible transition options that align individual competencies with healthcare system needs.

Several limitations of implementing structured retraining programs warrant consideration. First, the development and maintenance of such programs require substantial resource investment from healthcare institutions and professional organizations. Second, the effectiveness of transition programs may vary depending on individual surgeons' baseline competencies in primary care and their adaptability to new roles. Additionally, the success of such programs depends heavily on the availability of experienced mentors willing to guide transitioning surgeons through their career change.

## 4 Conclusion

The management of aging surgeons requires a balanced approach that considers both patient safety and the valuable experience these practitioners possess. While age-related decline varies individually, evidence suggests that chronological age alone should not determine surgical career endpoints. The Japanese experience demonstrates how surgeons can successfully transition to alternative roles, particularly in primary care, when supported by healthcare systems that embrace integrated clinical practice. This model, combined with structured assessment programs and transition support, could provide a framework for other high-income countries facing similar demographic challenges. As healthcare systems globally confront aging physician populations, developing evidence-based, standardized approaches to support career transitions while maintaining quality of care becomes increasingly crucial.
